# Characteristics and Key Genetic Pathway Analysis of Cr(VI)-Resistant *Bacillus subtilis* Isolated from Contaminated Soil in Response to Cr(VI)

**DOI:** 10.3390/toxics14010053

**Published:** 2026-01-04

**Authors:** Yiran Zhu, Peng Chen, Muzi Li, Qi Zheng, Jianing Li, Fuliang Zhang, Pimiao Zheng, Jianzhu Liu

**Affiliations:** 1 College of Veterinary Medicine, Shandong Agricultural University, Tai’an 271018, China; 2 School of Medicine, Jiangsu University, Zhenjiang 212003, China; 3 Research Center for Animal Disease Control Engineering, Shandong Agricultural University, Tai’an 271018, China

**Keywords:** Cr(VI), contaminated soil, *B. subtilis*, characteristic and genetic analysis, safety

## Abstract

With increasing industrialization, hexavalent chromium (Cr(VI)) is used in various metal smelting and other industries, which, in turn, causes hexavalent chromium pollution. This study aimed to investigate the characteristics of isolated *Bacillus subtilis* (*B. subtilis*) from high-Cr(VI) soils and to evaluate its safety. Genomic and transcriptomic analyses were performed to explore its Cr(VI) response mechanisms, and a mouse model (24 mice) was established to evaluate the safety of the bacterium at different concentrations. Key genetic findings showed that Cr(VI) exposure significantly up-regulated the *Spx* gene and down-regulated the *CtsR* gene—two critical transcriptional regulators involved in stress response and development that mediate Cr(VI) tolerance. Pathway analysis revealed that ribosome RNA, redox balance, protein biosynthesis, metabolism, and cysteine biosynthesis play a significant role in bacterial Cr(VI) resistance. In the in vivo experiment, it was observed that the small intestine (SI), liver, and spleen of the mice remained normal without any injuries. Different levels of the F3 isolate demonstrated the ability to resist colonization by digestive juices, as observed in the SI slides. Consequently, *B. subtilis* can endure high levels of Cr(VI) by regulating redox process genes, which makes it a potential candidate for further research in selecting safe, tolerant, and bio-remedial isolates for Cr(VI) treatment.

## 1. Introduction

Chromium (Cr), the seventh most abundant element in the world, exists in various oxidation states, ranging from divalent to hexavalent. Among these valence states, Cr(VI) exhibits high solubility, toxicity, and mutagenicity, making it a matter of significant concern for public health [1]. Due to its carcinogenic, mutagenic, and teratogenic properties, hexavalent chromium (Cr(VI)) is considered a critical factor contributing to health hazards [2].

The persistent issue of hexavalent chromium contamination in both water and soil presents a significant and enduring environmental challenge with far-reaching implications. Elevated concentrations of Cr(VI) are frequently encountered in industrial effluents and wastewater discharges, contributing to the contamination of surface water bodies [3]. Furthermore, Cr(VI) has the capacity to infiltrate the soil matrix, resulting in soil pollution [4]. Of particular concern is the contamination of agricultural soils, which may facilitate the uptake of toxic chromium by crops, thereby potentially introducing it into the food chain and posing health risks [5].

Contemporary approaches to mitigating Cr(VI) contamination in diverse environmental matrices, including soil, water, and wastewater, encompass a wide range of strategies [6]. These strategies include chemical treatments that utilize various reagents and chemicals, such as ferric chloride, calcium hydroxide, and zeolites, to either precipitate or reduce Cr(VI) to the less toxic Cr(III). The adsorption process involves the use of various materials, including activated carbon, graphene oxide, and metal oxides, as adsorbents to capture Cr(VI) ions from aqueous solutions [7]. On the other hand, nanomaterials, such as magnetite nanoparticles, nanotubes, and nanocomposites, play a pivotal role in enhancing Cr(VI) removal due to their unique properties [8]. Furthermore, microbial remediation employs bacteria and fungi with the capability to biologically reduce Cr(VI) to Cr(III) [2]. This microbial strategy has gained attention due to its cost-effectiveness and environmental friendliness. Various microbial species, including bacteria and fungi, have demonstrated the ability to perform this bioreduction, with specific enzymes like chromate reductases playing a crucial role [2,9]. Isolated from Cr(VI)-polluted habitats, *B. subtilis* can completely reduce toxic Cr(VI) to less hazardous Cr(III) even under alkaline conditions via constitutive membrane-bound enzymes—an activity further enhanced by humic acid, which boosts reduction efficiency and mitigates Cr(VI)-induced bacterial toxicity [10]. Complementing this direct bioreduction, the bacterium supports long-term soil remediation through dual mechanisms: regulating key stress-responsive genes to reinforce Cr(VI) tolerance and secreting extracellular polymeric substances that adsorb and immobilize reduced Cr(III), lowering its phytoavailability and thereby curbing chromium bioaccumulation in plants [11]. Microbial remediation offers a sustainable and promising approach for mitigating Cr(VI) pollution in contaminated soils and water bodies, contributing to environmental protection and human health [12,13].

From the perspective of microbial remediation, this study aims to characterize the Cr(VI) tolerance and reduction capacity of *B. subtilis* isolated from Cr(VI)-contaminated soil, explore the response mechanism of the strain (isolated from a high-Cr(VI) stress environment) from the perspective of probiotics alleviating intestinal stress, identify key genetic regulators and metabolic pathways underlying its Cr(VI) response via genomic analyses, evaluate the in vivo safety of the F3 isolate, and verify its potential for Cr(VI)-contaminated soil bioremediation. It is expected that this study will advance research on the microbial remediation of hexavalent chromium pollution.

## 2. Experimental Procedures

### 2.1. Reagents and Antibodies

An EasyPure Bacterial DNA Kit (Cat: EE161-01) was obtained from TransGen Biotech Co., Ltd. (Beijing, China). Muller–Hinton agar (MHA, Cat: HB6232), nutrient agar (NA, Cat: HB0109), nutrient broth (NB, Cat: HB0108), and biochemical tubes were received from Qingdao Hopebio Biology Technology Co., Ltd., Qingdao, China. Antibiotic-sensitive tablets were provided by Hangzhou Binhe Microbiological Reagent Co., Ltd., Hangzhou, China. K_2_Cr_2_O_7_ was obtained from Kaitong Chemical (Tianjin, China).

### 2.2. Isolation and Identification of B. subtilis from High-Cr(VI) Soils

The soil samples were acquired from a factory used to accumulate industrial chromium slag in Shenyang, Liaoning province, China (Figure S1). Initially, ten high-Cr(VI) soil samples (T_1_-1 to 5 and T_2_-1 to 5) were dried in an incubator at 80 °C for 1 h. *B. subtilis* endospores tolerate this temperature, while labile non-spore-forming microbes are inactivated, reducing isolation competition [14]. After 10 g of soil was added to 90 mL sterilized water, the mixtures were shaken for 30 min. The diluted cultures were isolated and incubated using NA medium at 37 °C for 24–72 h. The suspected dominant colonies were purified and 16S rRNA sequencing and biochemical tests were performed.

Genomic DNA was extracted using an EasyPure Bacterial DNA Kit (TransGen Biotech Co., Ltd., Beijing, China) according to the manufacturer’s manual. Bacterial DNA was amplified with universal primers 27F (5′-AGAGTTTGATCCTGGCTCAG-3′) and 1492R (5′-GGTTACCTTGTTACGACTT-3′). Subsequently, the PCR products were sequenced by Sangon Biotech (Shanghai) Co., Ltd, Shanghai, China. and then aligned using the National Center of Biotechnology Information (NCBI) BLAST+ 2.17.0. Then, phylogenetic trees were constructed via obtained and reference sequences using MEGA7 software [15]. The purified colonies were detected by biochemical tests involved in the bile biochemical tube test (Cat: GB157), D-mannitol biochemical tube test (Cat: GB179), propionate biochemical tube test (Cat: GB180), 7% NaCl biochemical tube test (Cat: GB181), nitrate biochemical tube test (Cat: GB182), D-xylose biochemical tube test (Cat: GB183), L-arabinose biochemical tube test (Cat: GB184), starch biochemical tube test (Cat: GB185), and pH 5.7 biochemical tube test (Cat: GB186).

The susceptibility of the isolated strains was detected by nine types of antibiotics consisting of penicillin (10 µg, Cat: 1001), cefotaxime (30 µg, Cat: 1085), kanamycin (30 µg, Cat: 1030), streptomycin (10 µg, Cat: 1031), gentamicin (10 µg, Cat: 1028), tetracycline (30 µg, Cat: 1036), doxycycline (30 µg, Cat: 1037), erythromycin (15 µg, Cat: 1039), and lincomycin (2 µg, Cat: 1053). The fresh bacterial suspensions (approximately 10^8^ CFU/mL) were evenly coated on Muller–Hinton agar plates, and then antibiotic disks were placed on the agar plates. Bacilli were grown on agar at 37 °C for one day before the inhibition zones were measured.

### 2.3. Scanning Electron Microscopy (SEM) and Transmission Electron Microscopy (TEM) of B. subtilis

F3 and T2 isolates were treated with 160 mg/L K_2_Cr_2_O_7_ for 12 h at 37 °C, respectively. Washed bacteria with saline were fixed with an electron microscope fixative at room temperature for 2 h and then saved at 4 °C. Subsequently, the bacteria were dehydrated with a graded ethanol series. One portion of the strains was dried by a critical-point dryer (K850, Quorum Technologies Ltd., Laughton, East Sussex, UK) and then treated with a conductive procedure. The representative pictures were viewed by SEM (SU8100, Hitachi High-Tech Corporation, Tokyo, Japan). Another portion of the isolates was cut into ultrathin sections and then double-stained in uranyl acetate and lead. The related images were photographed by TEM (HT7700, Hitachi High-Tech Corporation, Tokyo, Japan). ImageJ 1.51 was used to measure the dimensions (length, *l* and width, *w*) on the SEM cell images. Based on Baldwin and Bankston’s reports, the bacteria were expected to be hemispherical cylinders whose surface area (*S*) and volume (*V*) were assessed using the formula *S* = 4Π(*w*/2)^2^ + 2Π(*w*/2)(*l* − *w*) and *V* = 4Π(*w*/2)^3^/3 + Π(*w*/2)^2^(*l* − *w*) [16].

### 2.4. Establishment of B. subtilis Model to Respond to High-Cr(VI) Stress

F3 strains with an inoculum size of 1% (*v/v*) were shaken in NB (Group 1) or NB containing 160 mg/L K_2_Cr_2_O_7_ (Group 2) at 37 °C for 12 h. The strains of group 1 were divided into two parts involving genome sequencing and RNA sequencing. All experiments were conducted with three independent biological replicates and three technical replicates per biological sample, unless otherwise specified in the corresponding subsections.

#### 2.4.1. Genome Sequencing, Assembly, and Annotation

Before sequencing, the isolation of group 1 genomic DNA was carried out using a Bacterial Genomic DNA Extraction Kit (Qiagen, Hilden, Germany) according to the manufacturer’s protocols. One microgram of genomic DNA was repaired and size-selected (approximately 10 kb). Genomic DNA sequencing was performed using the Ligation Sequencing 1D Kit (Oxford Nanopore, Oxford, UK) and then utilizing the PromethION DNA sequencer (Oxford Nanopore Technologies plc, Oxford, UK) to acquire the raw sequencing data. Raw read quality assessment was conducted using FastQC v0.11.9, with adapter trimming and low-quality read filtering (Q-score < 20) performed by Porechop v0.2.4 and Nanofilt v2.8.0, respectively. To improve assembly accuracy, Nanopore long reads were corrected with Illumina short reads (paired-end, 150 bp) using Unicycler v0.5.0, which integrates short-read error correction for long-read data, followed by de novo assembly with Flye v2.9.1. The complete genome was acquired after the assembly, correction, and optimization of quality control reads. The gene function annotation of group 1 isolates was performed in the COG, KEGG, GO, Refseq, Pfam, and TIGRFAMs databases. Subsequently, the genome circle map was assembled according to genome sequencing depth, GC distribution, GC skew, and genome structure data.

#### 2.4.2. RNA Isolation and Sequencing Analysis

According to the manufacturer’s instructions, total RNA was isolated from group 1 and group 2 with three biological replicates per group. The quality and integrity of the extracted RNA were assessed by an Agilent Bioanalyzer with an RNA 6000 Nano kit (Agilent Technologies, Berkshire, UK) and agarose gel, respectively. The TruSeq RNA Sample Preparation kit (Illumina, San Diego, CA, USA) was used for the reverse transcription of cDNA obtained by RNA with an Integrity Number (RIN) > 7.0 to acquire cDNA libraries as per the instructions. Moreover, the library fragments (350 bp) were selected to a template of PCR amplification after the conversion of cDNA overhangs to blunt ends, the adenylation of the DNA fragment 3′ ends, and the integration with Illumina PE adapter oligonucleotides. Ultimately, the resulting libraries with quality control were sequenced by the Illumina sequencing platform (HiSeqTM 2500, San Diego, CA, USA). All sequencing data were presented in FASTQ format and filtered to gain high-quality reads by removing reads containing adapters, reads with unknown nucleotides (N) accounting for >5%, and low-quality reads with a Q-score ≤ 20 covering >20% of the read length. All downstream clean reads were mapped to the reference genome provided by Hisat2 (v2.2.1, Daehwan Kim Lab, Johns Hopkins University, Baltimore, MD, USA) software. The fragments per kilobase million reads (FPKM) value was calculated to assess the transcript or gene expression by the normalization of mapped reads and transcripts.

### 2.5. Measurement of the Bacterium’s Safety

#### 2.5.1. Animal Treatments

A total of 24 male BALB/c mice, 20 ± 2 g body weight, were purchased from a commercial farm specializing in laboratory animal breeding (Shandong, Tai’an, China) and maintained in cages with feed (b18asic diet) and water (purified water) freely at 22 ± 2 °C for the study. All mice were allowed to acclimatize for seven days. The twenty-four mice were divided into three groups (N = 8) and treated with 10^5^ CFU/g (T1), 10^6^ CFU/g (T2), and 10^7^ CFU/g (T3) of *B. subtilis* for seven days by intragastric administration, respectively. This dosage gradient reflects common probiotic safety assessment ranges to examine dose-dependent responses [17]. All of the procedures and animal care methods mentioned in this study were approved by the Institutional Animal Care and Use Committee of Shandong Agricultural University (SDAUA-2019-007).

#### 2.5.2. Tissue Preparation

After anesthesia with the intramuscular injection of 50 mg/kg Zoletil 50 (Virbac, Carros, France), blood samples were acquired from the hearts of three randomly selected mice as replicates. The blood was separated after being left for 30 min at room temperature, and then centrifuged at 3000× *g* for 15 min to obtain serum. Subsequently, the mice were euthanized to rapidly acquire SI, liver, and spleen samples for further assays.

#### 2.5.3. Sample Histology

The samples were fixed in universal tissue fixative (Cat: G1101 and G1113 (FISH)), which was at least ten times the volume of the tissues. After dehydration in gradient alcohol solutions, paraffin blocks were cut into slides for histological examination. The sections underwent hematoxylin and eosin (HE) staining, fluorescence in situ hybridization (FISH), and Sirius scarlet staining. 

#### 2.5.4. Fluorescence In Situ Hybridization (FISH)

The Cy3-labeled antisense probes (5′ CY3-CTTCAGCACTCAGGTTCG-CY3 3′) of *B. subtilis* were designed and synthesized by Servicebio Technology Co., Ltd. (Wuhan, China). The sections were deparaffinized, dehydrated in gradient alcohol, and immersed in DEPC solution to inactivate RNase. The SI samples were treated with proteinase K solution (20 μg/mL) before the slices were pre-hybridized for 1 h. Subsequently, the sections were hybridized with FISH probe mixtures at 37 °C overnight. Following strict washing processes, the nuclei were stained with 4,6-diamidino-2-phenylindole (DAPI). The fluorescent images were visualized using an ECLIPSE CI fluorescent microscope (Nikon, Tokyo, Japan).

#### 2.5.5. Sirius Scarlet Staining of Liver

Deionized water was used to wash the deparaffinized sections, which were then added to Sirius scarlet solution for 8 min. The slides were cleared of excess solution with deionized water and left in an oven until the tissues had dried. Liver tissues were dried with absolute ethanol in this step. After sealing with neutral gum, detailed images were obtained with an Eclipse E100 (Nikon, Tokyo, Japan).

### 2.6. Statistical Analysis

Student’s *t*-test (two-group type) and one-way analysis of variance (ANOVA, another group type) were performed to assess differences using IBM SPSS Statistics (version 19). Table data analyses were performed using the mean ± standard deviation (SD) of the experiments. Differences were considered significant at *p* < 0.05.

## 3. Results

### 3.1. Distribution and Traits of Bacillus

The dominant flora varied across ten samples, with Bacillus species dominating in five samples. In terms of morphological features and 16S rRNA gene sequencing, the acquired dominant species were *B. subtilis*, *Paenibacillus*, and *Micrococcus yunnanensis*. In order to reveal the phylogenetic affiliation of nucleotide sequences, phylogenetic trees were constructed of nine *B. subtilis* isolates from T_1_-1, T_1_-3, and T_1_-4 soil samples (Figure S2). The biochemical characteristics of the *B. subtilis* isolates were determined as described above. All isolates were able to grow at 7% NaCl, pH 5.7, and 80 °C, but could not withstand bile conditions for 24 h. The isolates utilized D-mannitol, D-xylose, and L-arabinose and degraded starch, but were not able to decompose propionate. The N1, N3, T1, and T2 strains were capable of degrading nitrate, yet other isolates did not possess this capability (Table 1). In particular, *B. subtilis* strains N3 and T1 were highly sensitive (HS, 15–20 mm inhibition zone) to streptomycin; strains N4, T1, and F1 were moderately sensitive (MS, 10–15 mm inhibition zone) to lincomycin; and all other tested strains were extremely sensitive (ES, >20 mm inhibition zone) to the aforementioned antibiotics, in accordance with EUCAST v13.0 [18] guidelines (Table 2).

### 3.2. SEM and TEM of F3 and T2 Isolates

**Figure 1:**
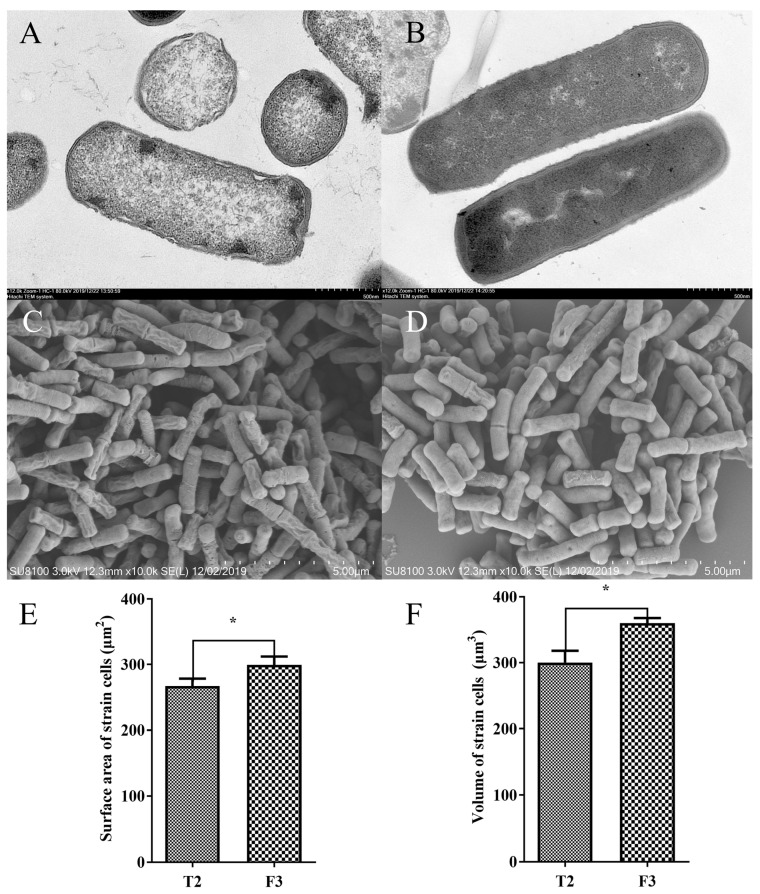
Morphology images and analysis of T2 and F3 strains after Cr(VI) exposure. Morphological changes of T2 (**A**) and F3 (**B**) strains captured by TEM. Images of T2 (**C**) and F3 (**D**) strains obtained by SEM. The surface area (**E**) and volume (**F**) of T2 and F3 strains were analyzed using SEM images. Data expressed as mean ± SD; “*” indicates significant difference (*p* < 0.05).

As illustrated in Figure 1, the cells of the F3 and T2 isolates stressed in a high Cr(VI) environment exhibited a high degree of morphological damage. Overall, the bacterium cells were coryneform, the cell wall became coarse, the organelle structure appeared fuzzy, and the protoplast increased in concentration. The cell morphology of the T2 and F3 cells changed, with the cell membrane transforming into an irregular shape to tolerate the Cr(VI) treatment (Figure 1A,B). The bacterium cells of the F3 isolate were longer than the T2 cells and exhibited irregular shapes and coccoid-like forms, as shown in Figure 1A,C. Meanwhile, the degree of shrinkage in the T2 cells was more obvious than in F3 cells (Figure 1C,D). Under high Cr(VI) pressure, the surface area and volume of the F3 cells were greater than those of the T2 cells (Figure 1E,F).

### 3.3. Genome Sequencing

**Figure 2:**
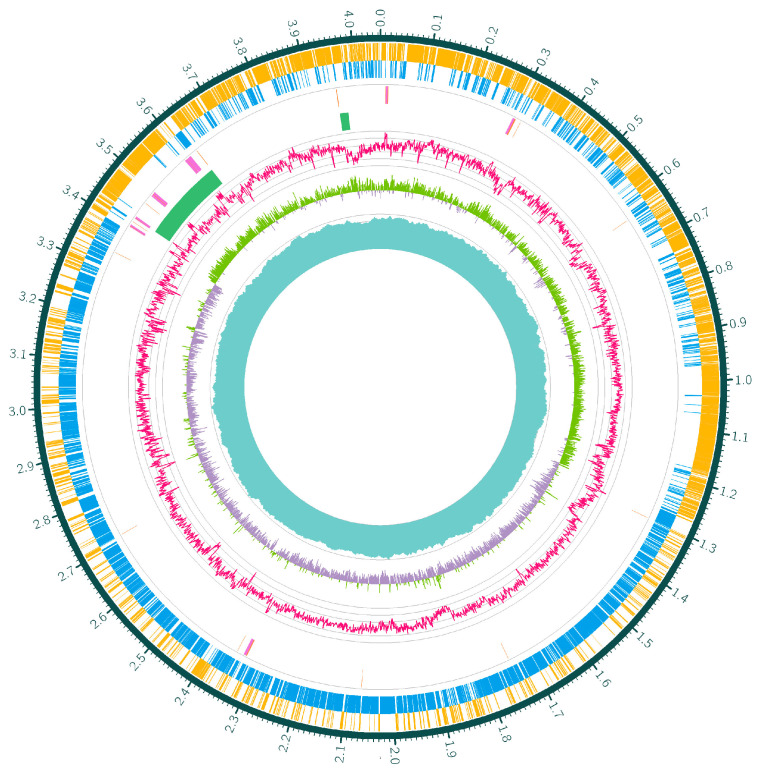
Genome circle map of the *B. subtilis* F3 isolates. The circular representation includes coding genes (sense strand and negative sense strand), tRNA (orange), rRNA (purple), CRISPR (blue), gene island (green), G + C ratio, GC skew, and genome sequencing depth from the outmost circle inwards.

**Figure 3:**
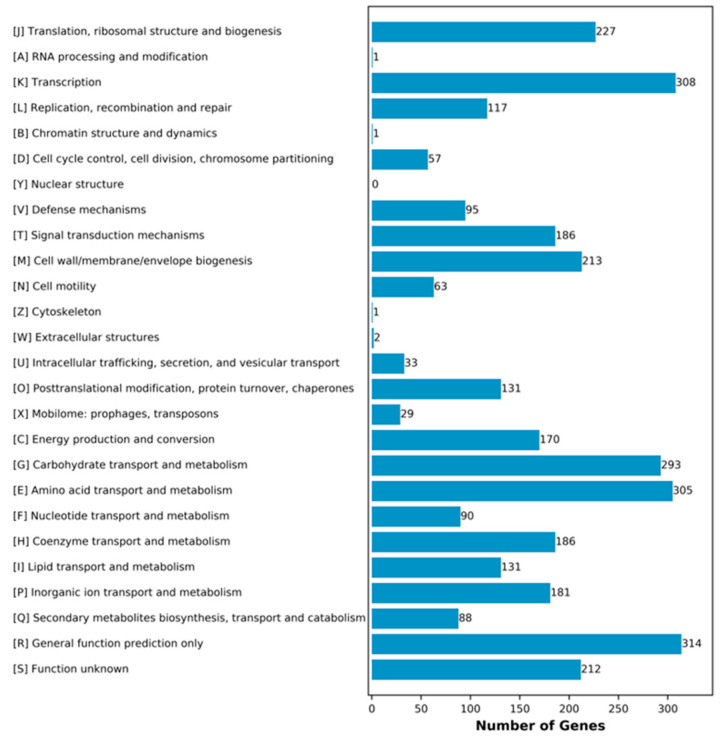
Classification statistics of COG functions of genome-encoded proteins in *B. subtilis* F3 isolates.

Given its more typical and stable Cr(VI) stress response phenotype among the representative isolates, F3 was selected for genome sequencing to elucidate the core genomic organization underlying *B. subtilis*’ survival under Cr(VI) stress. Initially, it was proven that the F3 strain was *B. subtilis* after genome sequencing analysis. The circular features of the F3 isolate are illustrated in Figure 2, in which the complete genome consists of 4,054,239 bp contig chromosomes. Genome sequencing revealed an average GC content of 43.60%, 4160 coding sequences (CDSs), a tRNA value of 86, and an rRNA value of 30 (Table 3). Moreover, 71.01% of the CDSs (2954) were functionally annotated to 26 clusters of orthologous groups (COGs), in which a considerable number of genes were linked to general function prediction, transcription, and metabolism pathways such as amino acid transport, carbohydrate transport, translations, ribosomal structure, and biogenesis as well as cell wall, member, and envelope biogenesis (Figure 3).

### 3.4. RNA Sequencing

To probe the response of *B. subtilis* F3 on Cr(VI), the RNA-Seq technique was applied to identify expression profiles with the Illumina HiSeq^TM^ 2500 system (Illumina, San Diego, CA, USA). Initially, the average of the total reads for each sample was 9,519,817. Furthermore, quality control was performed to acquire the average sequencing data containing clean reads of 9,485,371, percentage of clean reads of 99.64%, clean bases of 1.42 Gb, GC content of 44.92%, Q20 of 98.12%, and Q30 of 94.08% (Table 4). Clean reads were obtained after abandoning 0.36% of raw reads for later analysis. Additionally, the average map rate of clean reads in the abovementioned genome was found to be about 97.90%.

A total of 1378 differential genes were identified after *B. subtilis* F3 responded to Cr(VI) exposure—649 up-regulated and 729 down-regulated genes—among which 1360 genes possessed definite annotation information (Table S1). Under Cr(VI) stress, the expression of multiple functional genes in *B*. *subtilis* isolate F3 was significantly altered (Table 5). Specifically, genes involved in cold shock response (*Csp*), transcriptional regulation *(Spx*, *SenS*), beta-lactam resistance, cysteine transmembrane transport, metal ion binding, cysteine anabolism, and bacterial regulatory pathways (*gntR family*, *tetR family*, *anaerobic regulation*) were up-regulated. In contrast, several other genes showed decreased expression, including two beta-lactam resistance genes, numerous transcriptional regulator genes (*CtsR*, *vrH*, *LevR*), genes related to cysteine metabolism (*homocysteine S-methyltransferase*, *MetC*), and genes encoding cytochrome ubiquinol oxidase subunits, cytochrome c oxidase assembly factors, ubiquinol–cytochrome c reductase complex components, and cytochrome bd terminal oxidase subunit I.

Gene Ontology (GO) functional enrichment analysis was performed to reveal the topGO results for differential genes, of which the three main branches were biological process (BP), cellular component (CC), and molecular function (MF). Overall, differential genes were mainly enriched in the ATP catabolic process, protein phosphorylation, the hydrogen peroxide biosynthetic process, the transmembrane receptor protein tyrosine kinase signaling pathway, actin nucleation, sister chromatid cohesion, the cysteine biosynthetic process, protein deSUMOylation, and rRNA processing (Table 6). The data indicated that the small-molecule metabolic process and cellular metabolic process were the most enriched functions in the differential genes in BP parts; the ribosomal subunit and ribosome were more enriched in CC courses; and differential genes were clustered in the structural constituents of ribosome and structural molecule activity in MF processes.

**Figure 4:**
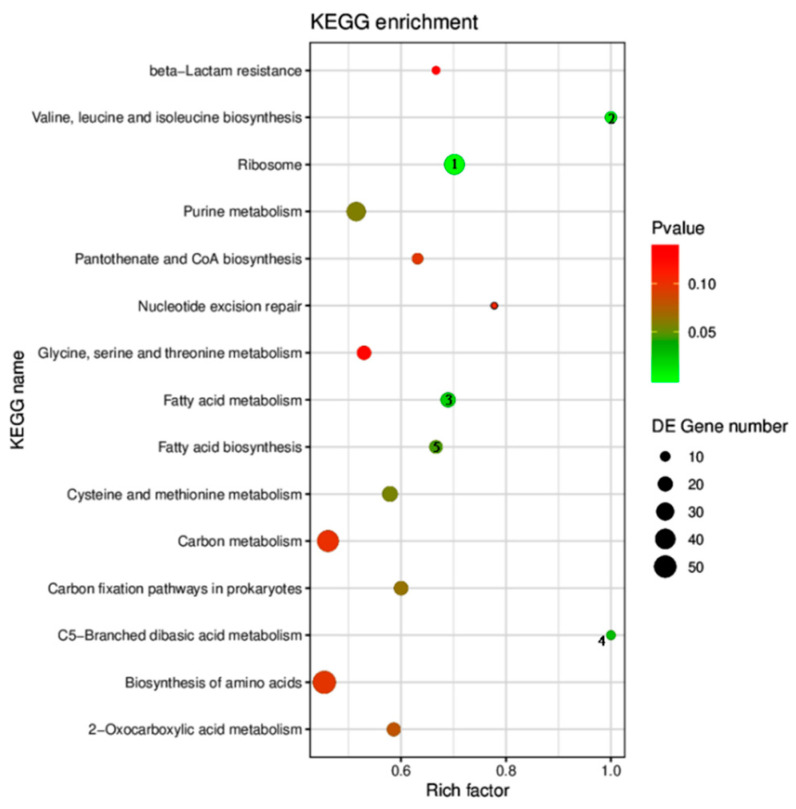
Enrichment pathways of differentially expressed genes in the KEGG database. Pathways with greater reference values are represented by redder color and larger horizontal axis. The displayed results are the pathways with the most reliable enrichment significance (namely, the smallest q value). The numbers on the circles represent the identification codes of the corresponding KEGG pathways (used for traceability to the original KEGG database entries). Specific information regarding the numbered pathways is provided in Table 7.

The pathway analysis of the differentially expressed genes revealed that 108 pathways were enriched after Cr(VI) treatment based on the KEGG database (Figure 4). The top 15 pathways with the most reliable enrichment significance included ribosome, valine, leucine, and isoleucine biosynthesis; fatty acid metabolism; C5-branched dibasic acid metabolism; fatty acid biosynthesis; cysteine and methionine metabolism; purine metabolism; carbon fixation pathways in prokaryotes; 2-oxocarboxylic acid metabolism; pantothenate and CoA biosynthesis; biosynthesis of amino acids; carbon metabolism; nucleotide excision repair; and glycine, serine, and threonine metabolism (Figure 4). In detail, the minimum q value (corrected *p*-value) was found for the ribosome pathway, whereas the maximum number of enriched genes was found for the biosynthesis of amino acids pathway. Further crucial pathways enriched after Cr(VI) exposure with *p*-values <0.05 included ribosome, valine, leucine, and isoleucine biosynthesis; fatty acid metabolism; C5-branched dibasic acid metabolism; and fatty acid biosynthesis (Table 7).

### 3.5. Safety Assessment of B. subtilis

#### 3.5.1. HE Staining

**Figure 5:**
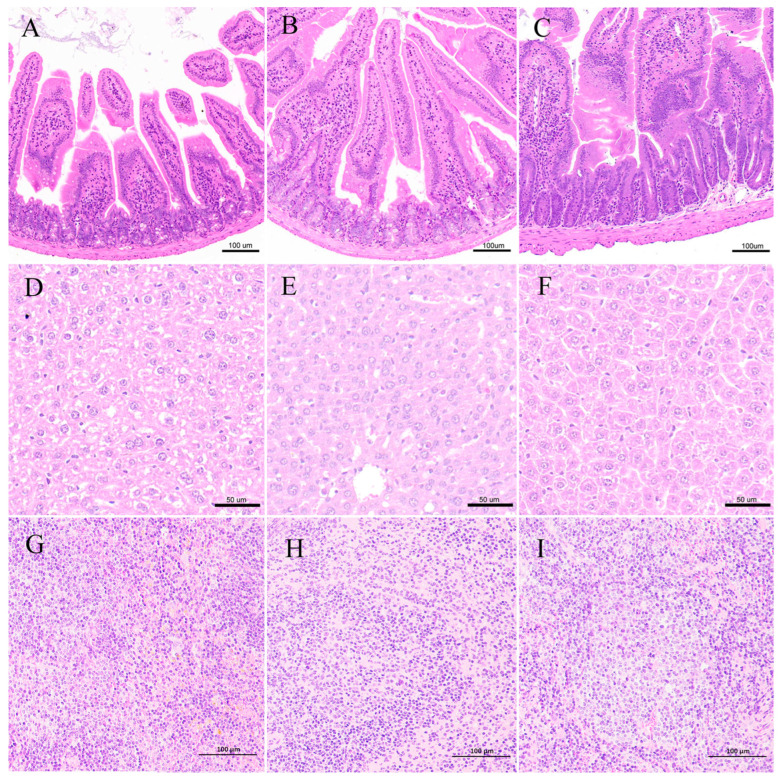
HE staining of mouse SI (**A**–**C**), liver (**D**–**F**), and spleen (**G**–**I**). Images are of the T1 (**A**,**D**,**G**), T2 (**B**,**E**,**H**), and T3 (**C**,**F**,**I**) groups.

In all three groups, the SI sections from the mice demonstrated that the mucosa and microvilli were intact, with no inflammatory cell infiltration and lymphatic expansion (Figure 5A–C). The basic architecture of the liver cells remained intact, exhibiting clear hepatic lobules, regular-arranged hepatocytes, and the absence of liver damage (Figure 5D–F). The HE staining of the structure of the spleen revealed normal and obvious white pulp and red pulp. Lymphocytes were tightly arranged in the T1–T3 groups (Figure 5G–I).

#### 3.5.2. FISH

**Figure 6:**
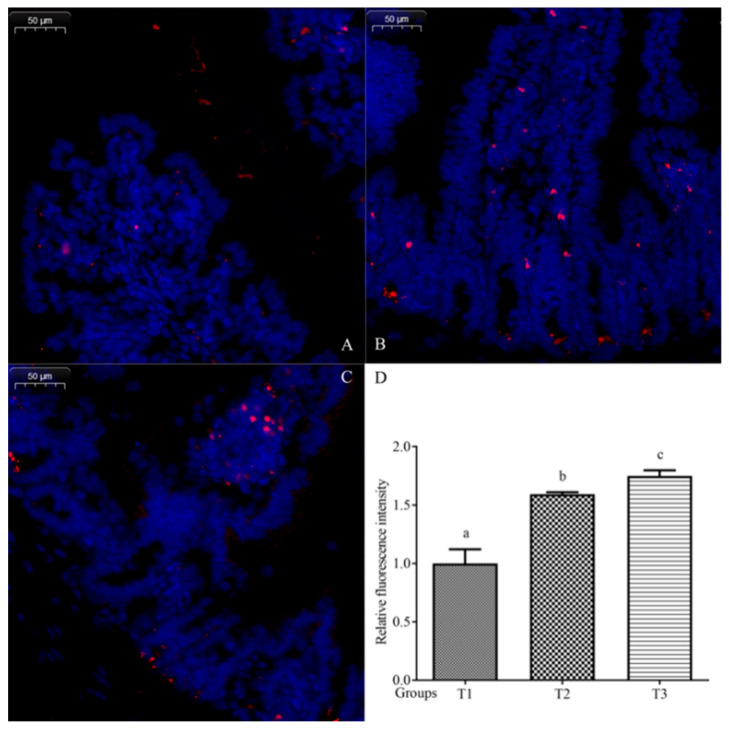
Fluorescence and analysis images of FISH staining in *B. subtilis*. Images are of the T1 (**A**), T2 (**B**), and T3 (**C**) groups. The quantifications of relative fluorescence were assessed in three groups (**D**). Data expressed as mean ± SD; different superscripts (a, b, c) indicate significant difference (*p* < 0.05).

The SI sections were stained with the *B. subtilis* probe depicted in Figure 6. The fluorescence images of red *B. subtilis* and blue gut cell nuclei revealed the colonization regions of *B. subtilis* in the intestinal mucosa. *B. subtilis*-positive areas were found from groups T1 to T3. Among the three groups, T3 presented the most positive fluorescence regions. FISH fluorescence images of the three different concentrations of *B. subtilis* are illustrated in Figure 6A–C. The fluorescence intensity of group T1 was considered standard, with the other groups expressed as the fluorescence intensity relative to T1. The positive fluorescence intensity in the T2 and T3 groups was almost 1.59 and 1.75 times that of the T1 group, respectively (Figure 6D).

#### 3.5.3. Sirius Scarlet Staining

**Figure 7:**
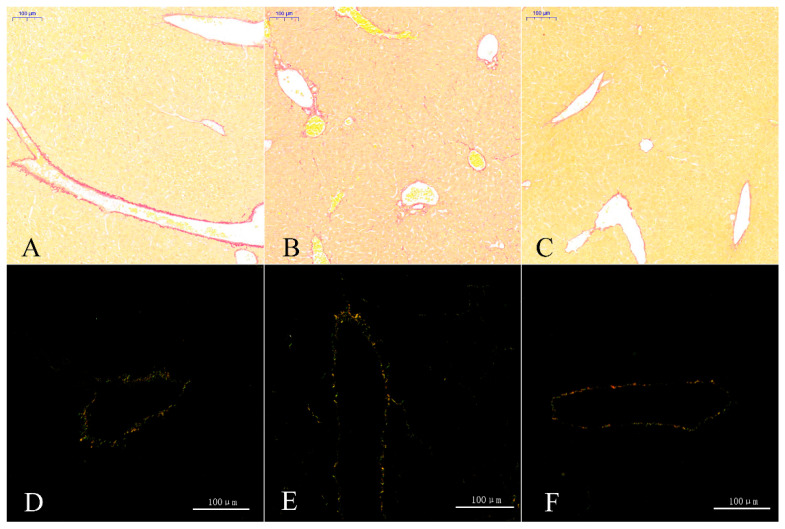
Sirius scarlet staining of liver cells. The images are of groups T1 (**A**), T2 (**B**), and T3 (**C**) under normal light and of groups T1 (**D**), T2 (**E**), and T3 (**F**) under polarized light.

In the in vivo experiment, Sirius scarlet staining of liver tissues showed no obvious collagen fiber proliferation across the three groups under normal light (Figure 7A–C), with only sparse red-stained collagen fibers observed in all groups. The collagen fibers were absent of distinct diversification according to a polarized light image analysis, mainly involving type I (red or yellow) and III (green, Figure 7D–F). These results indicate that, in the tested conditions, liver collagen fiber content and subtype composition remained relatively consistent across the three groups.

## 4. Discussion

### 4.1. Significance of Cr(VI) and the Role of B. subtilis in Remediation

Heavy metal contamination poses significant risks to both humans and animals, making it a subject of great concern. Efforts have been focused on addressing this issue by either removing toxic heavy metals from the soil or finding ways to immobilize them and prevent further pollution caused by their migration [19]. One effective method for remediating metal pollution in soils is microbial remediation, which involves the use of certain microorganisms, as previously demonstrated [20]. In this study, dominant Bacillus species were isolated from soil contaminated with Cr(VI). The nine Bacillus isolates were initially tested using three different biochemical tubes.

The first group was a tolerant reagent, involving bile and 7% NaCl at pH 5.7. The other two groups were carbohydrates consisting of D-mannitol, D-xylose, L-arabinose, and starch, as well as inorganic salts including propionate and nitrate, respectively. The strains *B. subtilis* T1, T2, N3, and N1 showed positive results in the nitrate biochemical tube test, distinguishing them from the other isolates. In order to prevent the emergence of antibiotic-resistant strains [21], it is important to conduct antibiotic susceptibility tests, as previous research has indicated. In the antibiotic tests performed in this study, the N3, N4, T1, and F1 isolates demonstrated moderate sensitivity to antibiotics. By selecting isolates that are extremely sensitive to antibiotics, the transmission and mutation of antibiotic resistance genes in bacteria can be prevented. Based on the phylogenetic trees, biochemical tube test results, and antibiotic susceptibility findings, the most distinct isolates of *B. subtilis* were identified as F3 and T2, which were selected for further study.

### 4.2. Morphological and Biochemical Characteristics of the Isolates

SEM and TEM are widely used techniques for visualizing the ultrastructure of bacterial cells. In general, bacterial cells possess a flexible surface area that allows them to tolerate various stressful conditions, including nutrient deprivation. This expanded surface area enables these cells to efficiently acquire nutrients, as previously demonstrated [22]. In the case of the F3 and T2 strains of *B. subtilis*, it was observed that the surface area and volume of the F3 strain were higher than those of the T2 strain in response to Cr(VI). This suggests that the F3 strain may have an advantage in obtaining nutrition. Based on these morphological features and the response to Cr(VI) exposure, the prominent *B. subtilis* strain F3 was selected for further investigation in this study.

### 4.3. Genomic and RNA-Seq Results—Adaptive Mechanisms

Research has shown that acquired adaptive solutions are related to the process of ingestion, absorption, reduction, and excretion of toxic metals that are beneficial to bacterial species survival in Cr(VI)-exposed soils [2]. As the characteristics of Bacillus members can adapt to harsh ecological environments to a certain degree, it was advantageous to identify the survival mechanism by revealing the bacterial genome sequences and gene response of F3 strains isolated from high Cr(VI) soil in this study. A total of 1360 genes were found to participate in the bacteria’s response to Cr(VI) stress. As previously mentioned, the CspA gene plays a crucial role in *Enterobacter ludwigii* LY6 strain’s reaction to cadmium stress [23] and increases under Cr(VI) stress, a finding that is consistent with the results in this study. *Spx* is a transcription regulator that plays conservative roles in low-GC Gram-positive bacteria, which can dominate varieties of stress response genes by strengthening and recognizing promoters. Its functions are not only reflected in the adjustment of stress response genes, but also in the intervention of developmental processes [24]. In addition, *Spx* revises translation through a complicated mechanism by inhibiting transcription ribosomal RNA and connecting with the stress process [24]. *CtsR*, as a repressor protein, can react to toxic stress diametrically and can be regulated by a negative feedback path [25]. In the present study, *Spx* gene expression was up-regulated and *CtsR* gene expression was down-regulated after Cr(VI) exposure. Furthermore, *Spx* was found to dominate gene expression by directly combining with the C-terminal domain of the RNA polymerase alpha subunit to activate or inhibit the transcription gene containing genes related to redox balance, protein regulation, and cysteine synthesis [26]. In the present study, the overwhelming majority of transcriptional regulatory proteins decreased, including CitT, YhcZ, DesR, LevR, YvrH, YvfU, DegU, YxjL, and NatR, yet only the expression of SenS increased. Moreover, there were two reactions in response to *Spx* activity: one was the acquisition of resistance to antibiotics, and the other was the tolerance of stressors [27]. *Spx* mainly promotes antibiotic resistance by regulating the cell wall stress response, as it is induced under cell wall stress, and its regulators play an adaptive role in combating antibiotics targeting peptidoglycan synthesis [28]. This is consistent with research finding that *Spx* can confer high levels of β-lactam resistance to Gram-positive bacteria and reduce sensitivity to first-line antibiotics. Accumulated *Spx* enhances resistance to cell wall- and membrane-targeted antibiotics, regardless of the redox sensing switch [29]. The beta-lactam resistance genes of CPEAOFNH_00182 (gene ID) increased, while CPEAOFNH_00476 (gene ID) and CPEAOFNH_03819 (gene ID) decreased in the current stage. Low-molecular-weight metallothioneins (MTs) consisting of plentiful cysteine as a metal-binding protein formed, which may participate in metal balance and protect cells from the toxicity of metals. The present study found this to be an indispensable role in cysteine biosynthesis after Cr(VI) stress. The cytochrome ubiquinol oxidase subunit—respiratory regulatory proteins—can avoid heavy-metal-induced oxidative damage by adjusting respiration in bacterial cells to reduce excessive ROS production [29]. This study confirmed the decrease in related cytochrome oxidase genes after Cr(VI) treatment, involving multiple genes such as cytochrome c oxidase subunits and assembly genes. Cytochrome bo3 is an essential respiratory oxidase of the heme–copper superfamily, and the related hydroquinone oxidase originated from cytochrome c oxidase [30]. In this study, Cr(VI) exposure decreased the cytochrome bo3 ubiquinol oxidase activity gene and the cytochrome ubiquinol oxidase subunit I gene, while the anaerobic regulatory protein gene increased, which is consistent with previous conclusions. The enrichment of the ATP catabolic process and protein phosphorylation in KEGG pathways indicated that these pathways may be involved in reacting to Cr(VI) stress.

### 4.4. Probiotic Potential and In Vivo Studies

Due to their broad antimicrobial ability and effective adhesion, most of the Bacillus strains are denoted as probiotic bacteria, especially *B. subtilis* [31,32]. The *B. subtilis* strains in this study were isolated and identified from available Cr(VI) soil resources and are widely used in dietary supplementation. The functional role of *B. subtilis* is based on its tolerance to low-acidic and bile-rich environments. Withstanding the stressful environment of gastrointestinal conditions was also one of the factors assessed to determine the viabilities of *B. subtilis*. Generally, simulated gastric and intestinal environments are required for evaluating effective tolerance, as previously indicated [33,34]. Bile salts provide a stressful environment to control the growth of bacteria [35,36], but tolerance to bile salts is also a necessary trait for probiotic bacteria. Furthermore, bacteria adjacent to the host areas can be monitored by FISH probe [37], which was applied in this study. In the current study, the discrepancies in *B. subtilis* colonization behaviors in animal experiments (T1–T3 groups) were displayed by the FISH probe. HE slides revealed tightly arranged hepatic cords, normal liver cell morphology, and a lack of inflammatory cell infiltration and collagen fiber deposition, indicating that the spleen’s histomorphological structure was not significantly different among T1–T3 groups without cell inflammation and injury. The FISH and histopathological examinations performed on the SI slides were effective in evaluating the animal models for cases of *B. subtilis* supplementation.

## 5. Conclusions

This study demonstrates the remarkable Cr(VI) tolerance of *B. subtilis* obtained from contaminated soil. Stress responses and key processes involving ribosomal RNA, redox homeostasis, protein metabolism, and cysteine regulation underpin its survival. Crucial tolerance genes have been identified in response to high Cr(VI) stress, laying a strong foundation for Cr(VI)-tolerant strain screening. Additionally, supplementation with *B. subtilis* is a promising protocol for bioremediation agents and Cr(VI) detoxification additives, as revealed by sequencing-based insights into its Cr(VI) response. These findings not only validate the strain’s potential for field-scale application in remediating Cr(VI)-contaminated soils, but also provide reference strategies for sustainable environmental restoration.

## Figures and Tables

**Table 1:** The biochemical tube test for isolates of *B. subtilis*.

Biochemical Tube Test	Isolates								
	N1	N2	N3	N4	T1	T2	F1	F2	F3
bile	−	−	−	−	−	−	−	−	−
D-mannitol	+	+	+	+	+	+	+	+	+
propionate	−	−	−	−	−	−	−	−	−
7% NaCl	+	+	+	+	+	+	+	+	+
nitrate	+	−	+	−	+	+	−	−	−
D-xylose	+	+	+	+	+	+	+	+	+
L-arabinose	+	+	+	+	+	+	+	+	+
starch	−	−	−	−	−	−	−	−	−
pH 5.7	+	+	+	+	+	+	+	+	+
80 °C growth	+	+	+	+	+	+	+	+	+

Note: +, positive; −, negative.

**Table 2:** The susceptibility of the isolated *B. subtilis*.

Susceptibility to Antibiotics	Isolates								
	N1	N2	N3	N4	T1	T2	F1	F2	F3
penicillin	ES	ES	ES	ES	ES	ES	ES	ES	ES
cefotaxime	ES	ES	ES	ES	ES	ES	ES	ES	ES
kanamycin	ES	ES	ES	ES	ES	ES	ES	ES	ES
streptomycin	ES	ES	HS	ES	HS	ES	ES	ES	ES
gentamicin	ES	ES	ES	ES	ES	ES	ES	ES	ES
tetracycline	ES	ES	ES	ES	ES	ES	ES	ES	ES
doxycycline	ES	ES	ES	ES	ES	ES	ES	ES	ES
erythromycin	ES	ES	ES	ES	ES	ES	ES	ES	ES
lincomycin	ES	ES	ES	MS	MS	ES	MS	ES	ES

ES: extremely sensitive, diameter of inhibition > 20 mm; HS: highly sensitive, 15–20 mm; MS: moderately sensitive, 10–15 mm.

**Table 3:** Sequencing data of the isolated *B. subtilis* F3 strain genome.

Feature	Value
Chromosome number	1
Genome size (bp)	4,054,239
GC content (%)	43.60
Protein coding genes (CDSs)	4160
rRNA	30
tRNA	86

**Table 4:** Sequencing data for each sample.

Sample	Total Reads	Clean Reads	Percentage of Clean Reads	Clean Bases	GC Content	% > Q20	% > Q30
Cr-1	9,099,616	9,066,906	99.64%	1,360,035,900	44.53%	98.05%	93.94%
Cr-2	10,259,728	10,226,208	99.67%	1,533,923,748	44.42%	98.12%	94.05%
Cr-3	10,661,038	10,622,664	99.64%	1,593,392,448	44.25%	98.12%	94.09%
F-1	9,565,790	9,537,602	99.71%	1,430,640,300	45.51%	98.27%	94.38%
F-2	8,621,352	8,595,512	99.70%	1,289,326,800	45.29%	98.20%	94.23%
F-3	8,911,376	8,863,334	99.46%	1,329,494,046	45.54%	97.98%	93.79%

Cr, *B. subtilis* F3 exposed to Cr(VI); F, *B. subtilis* F3 treated with nutrient broth.

**Table 5:** Gene expression patterns of *B. subtilis* isolate F3 under Cr(VI) stress.

Gene Category	Gene Name	Gene ID	Expression Pattern Under Cr(VI) Stress
Cold shock protein gene	*Csp*	CPEAOFNH_02613	Up-regulated
Transcriptional regulatory protein gene	*Spx*, *SenS*	CPEAOFNH_00606, CPEAOFNH_00312	Up-regulated
Beta-lactam resistance gene	*-*	CPEAOFNH_00182	Up-regulated
Cysteine transmembrane transporter gene	*-*	CPEAOFNH_00345, CPEAOFNH_00942, CPEAOFNH_04023	Up-regulated
Metal ion binding and phosphopantothenate-cysteine ligase gene	*CoaBC*	CPEAOFNH_01085	Up-regulated
Cysteine anabolic gene	*Prp*, *- (hydrolase)*, *- (synthase)*, *- (biosynthesis of L-cysteine from sulfate)*, *- (cystathionine)*, *- (selenocysteine)*	CPEAOFNH_02135, CPEAOFNH_03592, CPEAOFNH_02063/CPEAOFNH_03655, CPEAOFNH_02062, CPEAOFNH_03655, CPEAOFNH_03587	Up-regulated
Bacterial regulatory protein gene	*- (gntR family)*, *- (tetR family)*	CPEAOFNH_00338/CPEAOFNH_03465, CPEAOFNH_02787	Up-regulated
Anaerobic regulatory protein gene	*-*	CPEAOFNH_03180	Up-regulated
Beta-lactam resistance gene	*-*	CPEAOFNH_00476, CPEAOFNH_03819	Down-regulated
Transcriptional regulator protein gene	*CtsR*, *YvrH*, *LevR*, *NatR*, *DegU*, *YhcZ*, *YxjL*, *CitT*, *YvfU*, *DesR*, *Hpr*, *AlsR*	CPEAOFNH_03680, CPEAOFNH_02747, CPEAOFNH_02044, CPEAOFNH_03935, CPEAOFNH_02977, CPEAOFNH_00390, CPEAOFNH_03341, CPEAOFNH_00179, CPEAOFNH_02833, CPEAOFNH_01472, CPEAOFNH_00452, CPEAOFNH_03042	Down-regulated
Cysteine anabolic gene	*- (homocysteine S-methyltransferase)*, *MetC (cysteine lyase)*, *- (biosynthesis of L-cysteine from sulfate)*	CPEAOFNH_00554, CPEAOFNH_00646, CPEAOFNH_02771	Down-regulated
Cytochrome ubiquinol oxidase subunit	*- (cytochrome c), - (cytochrome c oxidase subunit I/II/III/IV)*	CPEAOFNH_00791, CPEAOFNH_01002/CPEAOFNH_01001/CPEAOFNH_01003/CPEAOFNH_01905/CPEAOFNH_01004	Down-regulated
Cytochrome c oxidase assembly factor gene	*CtaG, -*	CPEAOFNH_01005, CPEAOFNH_02213	Down-regulated
Ubiquinol–cytochrome c reductase complex gene	*-*	CPEAOFNH_01628, CPEAOFNH_01629, CPEAOFNH_01630	Down-regulated
Cytochrome bd terminal oxidase subunit I gene		CPEAOFNH_02456	Down-regulated

A hyphen (-) denotes the absence of homologous genes in the genome sequence of the target strain.

**Table 6:** TopGO enrichment results for differentially expressed genes.

GO.ID	Term	Annotated	Significant	Expected	KS
GO:0006200	ATP catabolic process	188	17	11.2	1.2 × 10^−17^
GO:0006468	protein phosphorylation	1416	79	84.38	8.2 × 10^−15^
GO:0050665	hydrogen peroxide biosynthetic process	96	9	5.72	1.90 × 10^−12^
GO:0007169	transmembrane receptor protein tyrosine kinase signaling pathway	136	14	8.1	2.5 × 10^−12^
GO:0045010	actin nucleation	182	6	10.85	2.80 × 10^−12^
GO:0007062	sister chromatid cohesion	140	4	8.34	1.70 × 10^−11^
GO:0019344	cysteine biosynthetic process	337	58	20.08	4.40 × 10^−11^
GO:0016926	protein deSUMOylation	89	4	5.3	4.80 × 10^−11^
GO:0006364	rRNA processing	328	57	19.55	9.50 × 10^−11^

**Table 7:** Top 5 crucial enrichment pathways with *p*-value < 0.05 after Cr(VI) exposure.

No.	Term	ID	Input Number	Background Number	*p*-Value	Corrected *p*-Value
1	Ribosome	ko03010	40	57	0.0011	0.1176
2	Valine, leucine, and isoleucine biosynthesis	ko00290	13	13	0.0090	0.4880
3	Fatty acid metabolism	ko01212	20	29	0.0210	0.7570
4	C5-branched dibasic acid metabolism	ko00660	9	9	0.0287	0.7762
5	Fatty acid biosynthesis	ko00061	16	24	0.0447	0.8671

## Data Availability

The original contributions presented in this study are included in the article/Supplementary Materials. Further inquiries can be directed to the corresponding authors.

## Supplementary Materials

The following supporting information can be downloaded at https://www.mdpi.com/article/10.3390/toxics14010053/s1, Figure S1: Soil sample collection location (a factory used to store industrial chromium slag in Shenyang, Liaoning province, China); Figure S2: 16S rRNA gene sequence similarity of *B. subtilis* isolates; Table S1: The annotation results of differentially expressed genes.
