# Supplementary material for: Characteristics and Key Genetic Pathway Analysis of Cr(VI)-Resistant Bacillus subtilis Isolated from Contaminated Soil in Response to Cr(VI)
Source: Toxics. 2026 Jan 4;14(1):53. doi: 10.3390/toxics14010053 (PMC12846181; doi:10.3390/toxics14010053)
Supplement: Supplementary file 1 [file toxics-14-00053-s001.zip › Figures Supplement.pdf]

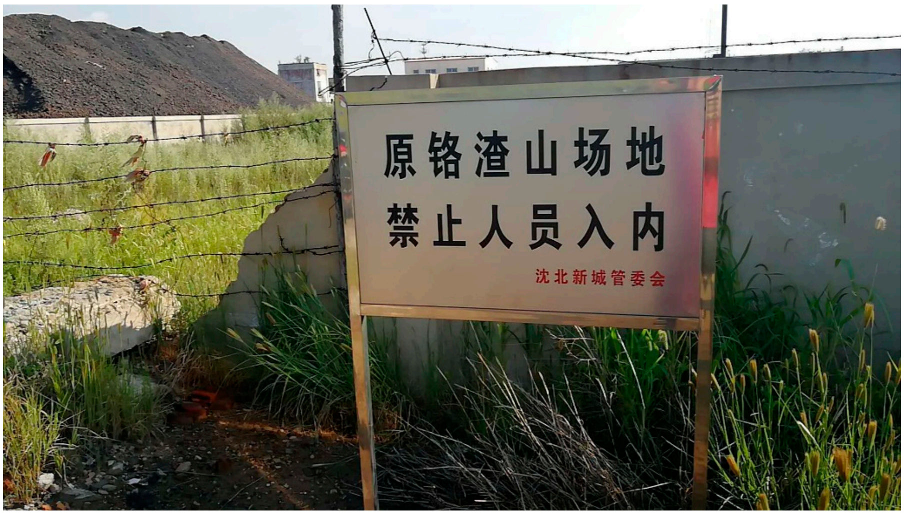

Fig. S1 Soil sample collection location（a factory used to accumulate industrial chromium slag in Shenyang, Liaoning province, China ）

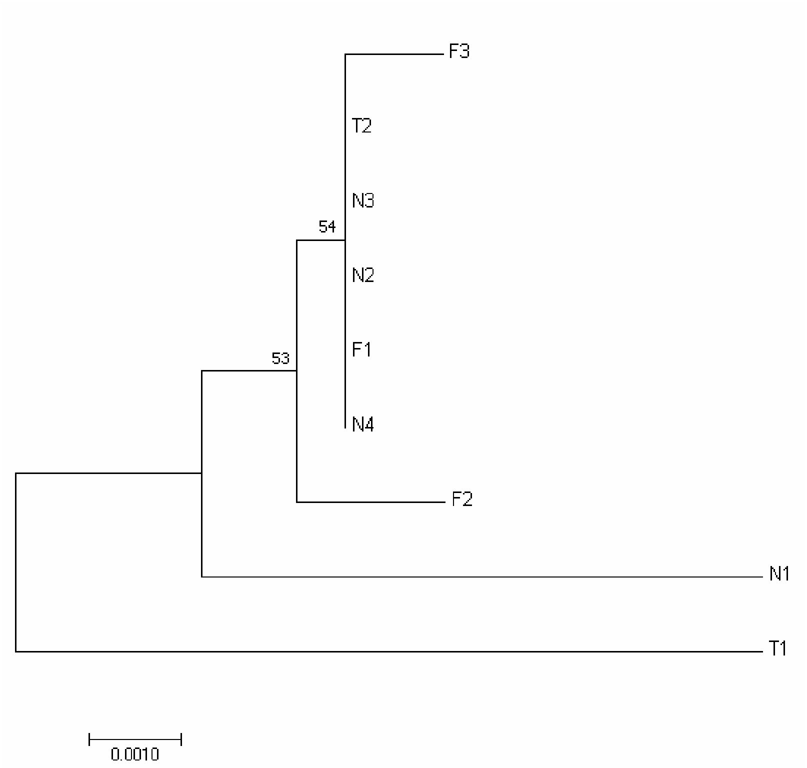

Fig. S2 16S rRNA gene sequence similarity of *B. subtilis* isolates.
